# Post-translational modifications on the retinoblastoma protein

**DOI:** 10.1186/s12929-022-00818-x

**Published:** 2022-06-01

**Authors:** Linbin Zhou, Danny Siu-Chun Ng, Jason C. Yam, Li Jia Chen, Clement C. Tham, Chi Pui Pang, Wai Kit Chu

**Affiliations:** 1 Department of Ophthalmology & Visual Sciences, The Chinese University of Hong Kong, Hong Kong, China; 2 Hong Kong Hub of Paediatric Excellence, The Chinese University of Hong Kong, Hong Kong, China; 3 Department of Ophthalmology & Visual Sciences, The Chinese University of Hong Kong, Hong Kong Eye Hospital, 147K Argyle Street, Kowloon, Hong Kong China

**Keywords:** Retinoblastoma, Phosphorylation, Ubiquitination, SUMOylation, Acetylation, Methylation

## Abstract

The retinoblastoma protein (pRb) functions as a cell cycle regulator controlling G1 to S phase transition and plays critical roles in tumour suppression. It is frequently inactivated in various tumours. The functions of pRb are tightly regulated, where post-translational modifications (PTMs) play crucial roles, including phosphorylation, ubiquitination, SUMOylation, acetylation and methylation. Most PTMs on pRb are reversible and can be detected in non-cancerous cells, playing an important role in cell cycle regulation, cell survival and differentiation. Conversely, altered PTMs on pRb can give rise to anomalies in cell proliferation and tumourigenesis. In this review, we first summarize recent findings pertinent to how individual PTMs impinge on pRb functions. As many of these PTMs on pRb were published as individual articles, we also provide insights on the coordination, either collaborations and/or competitions, of the same or different types of PTMs on pRb. Having a better understanding of how pRb is post-translationally modulated should pave the way for developing novel and specific therapeutic strategies to treat various human diseases.

## Background

**Fig. 1:**
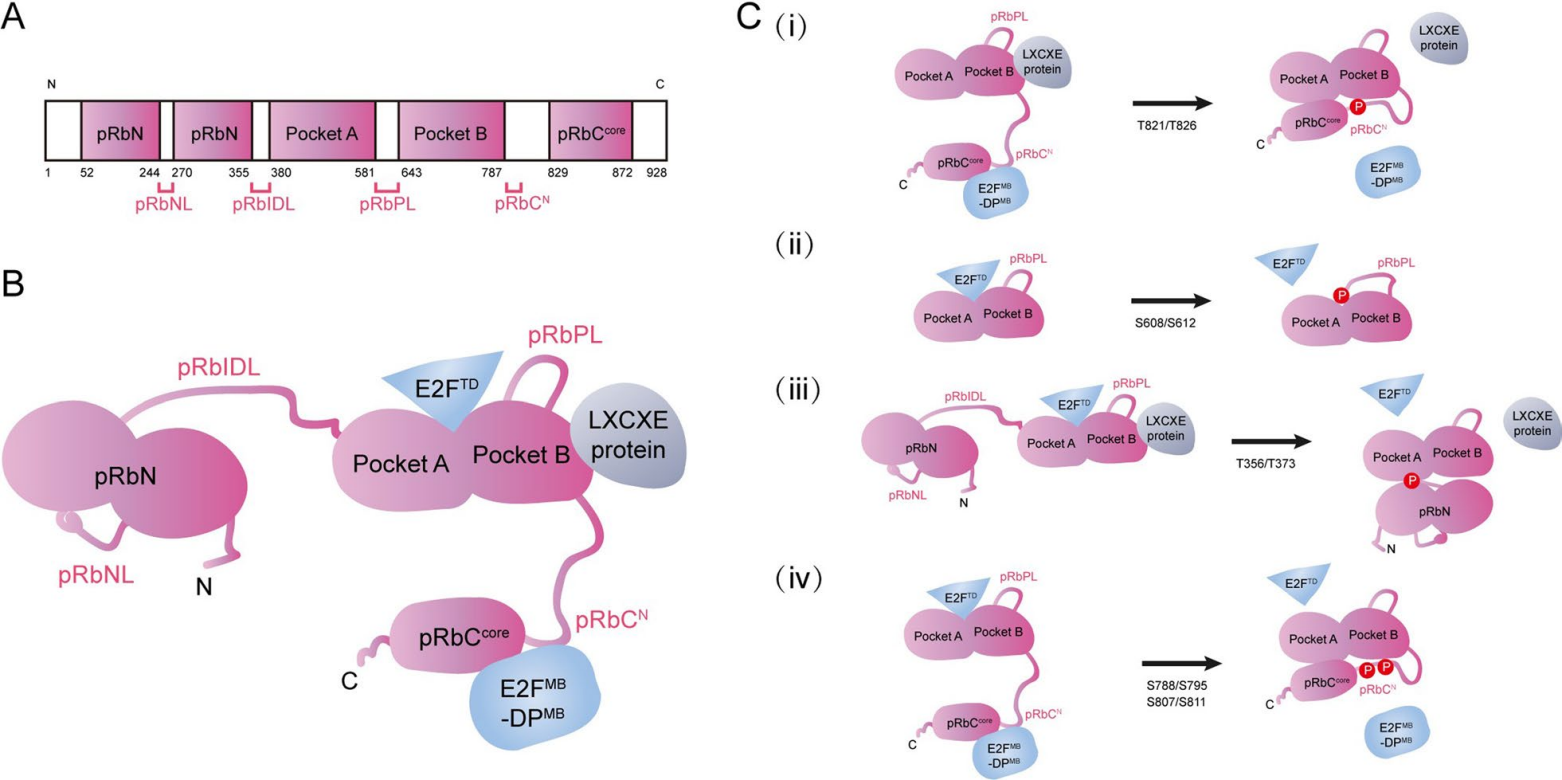
Retinoblastoma protein (pRb) structural domains and protein interactions. **A** Structured domains in pRb are colored, including the N-terminal domain (pRbN), the pocket domain A and B, and the pRb C-terminus core region (pRbC^core^). In contrast, several intrinsically regions contain two large loops in pRbN (pRbNL) and the pocket domain (pRbPL), an interdomain linker (pRbIDL) and part of the N-terminal region of the pRbC (pRbC^N^). N and C indicate the N- and C-terminals of the protein. Numbers indicate the amino acid positions. **B** Model of the unphosphorylated form of pRb and its interaction with E2F and LXCXE motif containing proteins. E2F^TD^ represents the E2F transactivation domain. E2F^MB^–DP^MB^ represents the marked box domains of E2F and its heterodimer partner DP. **C** Models demonstrating the impacts of various phosphorylation events on pRb structural alteration and on its association with E2F and LXCXE motif containing proteins. Only part of the pRb protein regions is shown for illustration. (i) T821/T826 phosphorylation promotes binding of pRbC^N^ to the pocket domain and inhibits pRb binding to LXCXE motif containing proteins as well as binding to E2F^MB^–DP^MB^. (ii) S608/S612 phosphorylation partially impedes E2F^TD^ interaction via promoting association of pRbPL with the pocket domain. (iii) T356/T373 phosphorylation partially blocks E2F^TD^ binding to pRb and pRb interacting with LXCXE motif containing proteins by inducing pRbN docking on the pocket domain. (iv) S788/S795 and S807/S811 phosphorylation facilitates intramolecular association between the pRbC and the pocket domain to obstruct the sites for E2F^TD^ and E2F^MB^–DP^MB^ binding

One well-acknowledged underlying etiology of cancer is a loss in cell proliferation control [1]. Regulation of G1 progression is an important step in cell proliferation control, and this process is highly sensitive to tumourigenesis [2]. Retinoblastoma susceptibility gene (*RB1*) is the first identified tumour suppressor gene. Its inactivation mutation was originally discovered as a cause of retinoblastoma in children [3]. On top of developing the retinal malignancy, retinoblastoma survivors are predisposed to acquire osteosarcoma and other sarcomas due to *RB1* inactivation mutation [4]. In normal eukaryotic cells, the tumour suppressive function of *RB1* is executed by its translational product, the RB protein (pRb) [5]. pRb is a DNA binding protein of 928 amino acids, containing two folded domains, a structured N-terminal domain (pRbN) and a central pocket domain, including the pocket A and pocket B domains. Both folded domains consist of two helical subdomains [6, 7]. Several intrinsically disorder sequences within pRb involve two loops in pRbN (pRbNL) and the pocket (pRbPL), an interdomain linker (pRbIDL) and parts of the C-terminal domain (pRbC) (Fig. 1A). The pocket domain of pRb binds its putative binding partner E2F transactivation domain (E2F^TD^) through a cleft between the two helical subdomains [8, 9]. Moreover, the pRbC also binds the marked box domains of E2F and its heterodimer partner DP (E2F^MB^-DP^MB^) [10]. Two regions of the pRbC, the N-terminal region of pRbC (pRbC^N^) and the core region of pRbC (pRbC^core^), are involved in this interaction [10]. On the other hand, the pocket B domain of pRb binds a linear LXCXE sequence motif in viral oncoproteins via its cleft (Fig. 1B) [6, 11].

pRb, together with its homologs p107 and p130, belongs to the “pocket protein” family and was first identified to serve as a cell cycle regulator to exert its tumour suppressive effects [12, 13]. To control cell proliferation, pRb tightly regulates the cell cycle checkpoint located in the G1/S phase boundary principally by governing the activities of the E2F transcription factor family members via direct binding or recruiting co-repressors [14]. In cancers with loss of the pRb function, *RB1* inactivation mutation or dysregulation of pRb upstream modulators constitutively inactivates pRb, giving rise to uncontrolled cell division [15–17]. In addition to its roles in G1 checkpoint control, pRb also plays crucial parts in many other cellular processes, involving differentiation, chromosomal stability, chromatin remodeling, angiogenesis, apoptosis and senescence [17].

Post-translational modifications (PTMs) are covalent attachments of functional groups to protein substrates and play critical roles in numerous biological processes. Thus far, more than 450 PTMs on proteins have been unveiled, inclusive of phosphorylation, ubiquitination, SUMOylation, acetylation and methylation. These PTMs are capable of altering the activity, stability, protein interaction and intracellular localization of the target proteins [18]. Most of the PTMs are reversible and changeable quantitatively without noticeable effects. They can also be drawn upon by normal eukaryotic cells as a “switch” to quickly alter cell states [19]. pRb is modified by various PTMs that can affect specific functions of pRb to maintain cellular homeostasis in specific contexts in normal eukaryotic cells [20]. Moreover, crosstalk between different PTMs on pRb is also finely tuned to accommodate normal eukaryotic cells to various changes in diverse settings and in specific circumstances [21–23]. Anomalies in PTMs can result in aberrant activities of pRb, responsible for dysregulated cellular processes such as oncogenesis [24, 25]. The main purpose of the present review is to comprehensively summarize the influence of ubiquitination, SUMOylation, phosphorylation, acetylation and methylation, on pRb functions under distinct physiological and pathological conditions. In addition, multiple PTMs have been detected on the same pRb molecules. How these multiple PTMs, either the same or different types of PTMs, are regulated on pRb and their impacts on the functions of pRb are also discussed.

## Main text

### (A) Ubiquitination of pRb

Ubiquitination, one important PTM, links ubiquitin, a protein with 76 amino acids, with a target substrate covalently in an ATP-dependent manner. The ubiquitination reaction involves multiple steps mediated by three types of enzymes: ubiquitin activating enzymes (E1s), ubiquitin conjugating enzymes (E2s) and ubiquitin ligases (E3s) [26]. Initially, E1s bind to ubiquitin along with ATP for activation, subsequently transfer the activated ubiquitin to E2s, and eventually the activated ubiquitin is covalently conjugated to the target residues on substrates by E3s [26]. One of the target residues that could be modified by E3s is lysine. Based on the amount of ubiquitin molecules conjugated to one lysine molecule on the substrate, ubiquitination can be categorized as monoubiquitination (single ubiquitin) and polyubiquitination (chains of ubiquitins) [27]. In polyubiquitination, the polyubiquitinated chain can be formed by ubiquitin attachment through the first methionine (M1) or 7 lysine residues (K6, K11, K27, K29, K33, K48, K63) [28]. Different polyubiquitination chains can lead to various consequences to the protein substrates, with K48-linked polyubiquitination engaged in both proteasomal degradation [29] and proteasome-independent regulation of signaling events and transcription [30, 31], while K11-linked polyubiquitination has been implicated in proteolysis [29] and K63-linked polyubiquitination has been reported in signaling convergence [29]. Throughout the entire process of ubiquitination reactions, E3s are particularly important as they serve to recruit specific substrates for ubiquitination. In human, E1s and E2s have 2 and 42 family members respectively, whereas several hundred of E3s have been identified thus far, which can be classified as the homology to E6AP C terminus (HECT) domain-containing E3s, the really interesting new gene (RING) finger domain-containing E3s and the RING-between-RING (RBR) family E3s [32, 33]. Ubiquitination processes can be reversed by deubiquitinating enzymes (DUBs), which can remove ubiquitin from ubiquitinated substrates [34]. Dynamic balance between ubiquitination and deubiquitination are tightly regulated to control cellular functions. Its deregulation could give rise to multiple diseases such as cancer [33].

pRb can be target of several E3 ligases (Table 1). These E3 ligases ubiquitinate pRb and promote its ubiquitin-dependent proteasomal degradation to affect its functions in cell cycle regulation. For example, tripartite motif containing 71 (TRIM71), an E3 ligase that was inactivated by phosphorylation via protein kinase A, ubiquitinated pRb and accelerated its degradation in a K48-linked polyubiquitination fashion, which facilitated breast tumor progression [35]. Moreover, RNF123, a member of the RING finger domain-containing E3 ligases, interacted with and mono-ubiquitinated pRb to mediate its degradation in cells expressing disease-causing Lamin A mutants, resulting in enhanced G1/S phase transition [36]. Novel RB ubiquitin E3 ligase (NRBE3) was found upregulated in breast tumour tissues and transcriptionally activated by E2F1/DP1 [37]. This E3 ligase selectively bound with the hypophosphorylated form of pRb through its LXCXE motif sequence and ubiquitinated preferably the hypophosphorylated pRb in a K48-linked polyubiquitination manner to destabilize pRb through proteasomal degradation, leading to accelerated G1/S phase transition and cell proliferation [37]. Additionally, human U3 protein14a (hUTP14a), a nucleolar protein with E3 ligase activity, interacted with pRb through PENF motif in its C-terminus, and polyubiquitinated pRb to increase its proteasomal turnover, which upregulated expressions of E2F1 regulated genes and enhanced proliferation of cancer cells [38].

**Table 1:** E3 ubiquitin ligases that target pRb for ubiquitination

E3 ligase	Adaptor(s)	Functional outputs	References
TRIM71	None	Facilitate breast tumor progression	[35]
RNF123	None	Enhance G1/S phase transition	[36]
NRBE3	None	Accelerate G1/S phase transition and cell proliferation	[37]
MDM2	None	Facilitate cell cycle progression	[24, 39, 41]
	MDMX	Restore pRb functions in cell cycle regulation [44] Enhance G1/S phase transition [45]	[44, 45]
	NIR	Elevate G1/S phase transition and cell proliferation [42]	[42]
SCF^SKP2^	EBNA3C	Attenuating pRb-induced G1 arrest	[25]
hUTP14a	None	Upregulate expression of E2F1 regulated genes and enhance proliferation of cancer cells	[38]
Cullin 2	HPV16 E7	Stimulate cell proliferation	[48]
E6AP	NS5B	Stimulate cell proliferation	[50]

Murine double minute 2 (MDM2), a putative E3 ligase for tumour suppressor p53 ubiquitination, also targets pRb for ubiquitination to exerts impacts on its functions in cell cycle regulation. And MDM2 is able to interact with pRb and affect its protein stability through diverse mechanisms. On one hand, MDM2 ubiquitinated pRb specifically but not the other pRb family proteins p107 and p130, for proteasomal degradation in a p53-independent fashion, reducing its protein stability and facilitating cell cycle progression [24, 39]. On the other hand, MDM2 interfered with pRb protein stability by interacting with pRb and C8 subunit of the 20S proteasome to promote its p53- and ubiquitin-independent proteasomal degradation, enhancing cell cycle S phase entry and DNA synthesis [40]. Besides, MDM2 can also modulate pRb protein level or stability in a cell cycle phase-dependent manner to govern cell cycle progression. For example, in G1 phase and under genotoxic stress conditions, MDM2 protein formed complex with the mRNA of pRb to guide it to polysomes to increase pRb protein synthesis, thereby contributing to G1 cell cycle arrest. Nevertheless, in the G2/M phase and upon genotoxic stress, MDM2 ubiquitinated and degraded pRb to promote cell cycle progression [41]. What signals or factors that direct pRb to degradation via specific pathways requires further investigation. Novel INHAT repressor (NIR), a nucleolar protein and novel histone acetyltransferase inhibitor (INHAT), was highly expressed in colorectal cancer tissues and significantly correlated with poor clinical outcome. It interacted with pRb via a LXCXE motif in its INHAT-2 domain and polyubiquitinated pRb for proteasomal degradation dependent on MDM2, giving rise to elevated G1/S phase transition and cell proliferation in colorectal cancer cells [42]. As NIR has not been reported to possess E3 ligase activity and can interact with MDM2 to inhibit MDM2 degradation [43], it would be interesting to further investigate whether NIR recruits MDM2 to form a ternary complex with pRb to promote pRb ubiquitination and degradation. MDMX, a structural homolog of MDM2 without detectable E3 ligase activity, competed with MDM2 for binding to the pRb C-terminal region, and impeded the MDM2-pRb interaction, leading to inhibitory MDM2-mediated pRb ubiquitination and degradation and therefore contributing to restoration of pRb functions in cell cycle regulation [44]. Contradictorily, another study showed that MDMX bound to the pRb C-pocket via its C-terminal ring finger domain and promoted MDM2-pRb interaction, causing pRb destabilization and inhibition of the suppressive activity of pRb on E2F1 in a MDM2-dependent fashion [45]. It is unknown whether this effect is dependent on ubiquitination. Meanwhile, the reasons for the discrepancy between these two observations on effects of MDMX on MDM2-pRb interaction are unclear and require further investigations.

Some E3 ligases can be recruited by viral oncoproteins without E3 ligase activity to ubiquitinate pRb and influence its functions in cell cycle regulation. Epstein–Barr nuclear antigen 3C (EBNA3C), an Epstein–Barr virus latency protein, interacted with pRb when the proteasome machinery was impeded, and recruited a ubiquitin ligase complex SCF^SKP2^, through its N-terminal 140–149 amino acids motif, to ubiquitinate and subsequently degrade pRb, but not the other two pRb family proteins p107 and p130, attenuating pRb-induced G1 arrest [25]. Apart from direct binding and subsequent sequestering pRb from E2F [46, 47], another DNA viral oncoprotein, human papilloma virus (HPV)-16 E7, was also able to associate with an active cullin 2 ubiquitin ligase complex via its elongin C subunit to polyubiquitinate and degrade pRb proteasomally [48]. In addition to DNA viral oncoproteins, RNA viral oncoproteins also affect cellular pRb abundance through ubiquitination. Nonstructural protein 5B (NS5B), a viral RNA-dependent RNA polymerase, enhanced pRb cytoplasmic localization by interaction with it via a conserved Leu-X-Cys/Asn-X-Asp motif, and further recruited an E3 ligase called E6-associated protein (E6AP) to target pRb for polyubiquitination and proteasomal degradation, which activated E2F-responsive promoters and stimulated HPV-infected hepatoma cell growth [49, 50].

Although site-specific regulations and functions of pRb ubiquitination have rarely been reported, several studies have revealed ubiquitination sites of pRb through high-throughput mass spectrometry (MS) under various conditions and in different cell types or tissues (Table 2). K803 on pRb was revealed to be ubiquitinated via TRIM71 in MMTV-Tg (LINK-A) mouse mammary gland tumour tissues by MS analysis [35]. In unperturbed HEK293T cells and in proteasome inhibitor MG132-treated MV4-11 cells, a K810 ubiquitination site of pRb was mapped [51]. Moreover, in unperturbed Jurkat E6-1 cells, up to 18 endogenous ubiquitinated sites on pRb were detected [52]. Apart from unperturbed condition, ubiquitination of pRb at specific sites were also discovered under stress conditions. For example, after ultraviolet (UV) treatment, ubiquitination of pRb at K842 in U2OS cells and at six sites (K879, K97, K856, K846, K823, K842) in HEK293T cells were found [53, 54]. Under a proteasome inhibition condition induced by bortezomid and b-AP15, 24 sites on pRb were identified to be ubiquitinated in Hep2 and Jurkat cells [55]. Besides, ubiquitination of pRb at K143 was unveiled in HCT116 cells with combined treatment of bortezomid and cycloheximide [56]. Intriguingly, ubiquitination of pRb at some identical sites (for example K810) seems to be common in some cell types regardless of being unperturbed or proteasome inhibition, suggesting that pRb ubiquitination at these sites probably plays similar roles in these conditions. On the other hand, ubiquitination of pRb at differential sites observed (for example K860, K432 and K879) might probably execute site-specific cellular functions in specific cell types under specific stress contexts.

**Table 2:** Ubiquitination sites of pRb detected by MS

Cell lines/tissues	Conditions	Ubiquitination sites detected by MS	References
MMTV-Tg (LINK-A) mouse mammary gland tumour	Untreated	K803	[35]
HEK293T cells; MV4–11 cells	Unperturbed (for HEK293T cells); proteasome inhibitor MG132 treatment (for MV4–11 cells)	K810	[51]
Jurkat E6-1 cells	Unperturbed	K63, K65, K265, K279, K289, K319, K329, K359, K420, K640, K791, K810, K814, K844, K847, K870, K896, K900	[52]
U2OS cells	UV treated	K842	[53]
HEK293T cells	UV treated	K97, K823, K842, K846, K856, K879	[54]
Hep2 and Jurkat cells	Treatment with proteasome inhibitors bortezomid and b-AP15	K63, K65, K94, K136, K143, K265, K279, K289, K327, K341, K420, K427, K432, K537, K640, K791, K810, K814, K824, K847, K870, K896, K900 (in Jurkat cells) K63, K65, K94, K136, K143, K265, K279, K289, K327, K341, K420, K427, K537, K640, K810, K847, K860, K870, K896, K900 (in Hep2 cells)	[55]
HCT116 cells	Combined treatment with bortezomid and cycloheximide	K143	[56]

### (B) Deubiquitination of pRb

In addition to ubiquitination, pRb is deubiquitinated with modulations on its functions. Herpes virus associated ubiquitin specific protease (HAUSP) deubiquitinated pRb and shielded it from K48-linked polyubiquitination and proteasomal degradation, resulting in increased pRb stability and subsequent G1 cell cycle arrest. The activity of HAUSP on pRb was subject to MDM2 in a context-specific fashion, where HAUSP deubiquitinated and stabilized pRb with low level of MDM2 in normal cells while high level of MDM2 hampered HAUSP activity on pRb leading to pRb degradation in cancer cells [57]. As MDM2 can target pRb for either ubiquitin-dependent or ubiquitin-independent proteasomal degradation [24, 40], MDM2 might potentially counteract HAUSP to affect pRb stability by either mechanism in cancer cells.

### (A) SUMOylation of pRb

SUMOylation is a PTM that modifies protein substrates with small ubiquitin-like modifiers (SUMOs) by adopting similar enzymatic mechanisms as in ubiquitination, where E1 activating enzymes, E2 conjugating enzymes and E3 ligases are engaged in catalyzing the covalent attachment of SUMOs to protein substrates [58]. In activation, the COOH termini of SUMOs are cleaved to conjugate with SUMO-activating enzyme (E1) supported by energy generated from ATP hydrolysis. Activated SUMOs are then transferred to UBC9, the only known SUMO-conjugating enzyme (E2). Subsequently, SUMOs form an isopeptide bond with specific lysine residues on protein substrates through SUMO ligases (E3s) [59]. In human, SUMOs have four distinct isoforms (SUMO-1, -2, -3 and -4). SUMO-1, SUMO-2 and SUMO-3 are the main SUMO proteins, among which SUMO-2 and SUMO-3 share 97% identity in terms of amino acid sequence, while SUMO-1 shares 50% sequence similarity with either SUMO-2 or SUMO-3 [60]. SUMO-1 is usually conjugated to a lysine residue of substrate as a monomer (mono-SUMO), while SUMO-2 or SUMO-3 forms a poly-SUMO chain (poly-SUMO). Besides, a substrate can be modified with SUMOs at multiple lysine residues (multi-SUMO) [61]. By covalent binding of these SUMO proteins to substrates at specific lysine residues, SUMOylation modulates cellular processes including DNA repair and synthesis, cell cycle regulation and subcellular localization [62–65]. Similar to ubiquitination, SUMOylation is reversible and controlled by the family of Sentrin-specific proteases (SENPs) via removing SUMOs from SUMO-conjugated substrates [61].

pRb can be SUMOylated to affect its functions in cell cycle regulation. On one hand, SUMOylation of pRb can promote cell cycle progression. For example, hypophosphorylated form of pRb was preferentially SUMOylated with covalent attachment of SUMO-1 at K720 to hinder suppressive effects of pRb on E2F transcription factor [66]. Furthermore, SUMOlyation of pRb at K720 by SUMO-1 occurred preceding its phosphorylation at the early G1 phase to recruit CDK2, which contains a SUMO-interaction motif (SIM), to promote pRb phosphorylation at S807/S811, leading to E2F1 release from pRb–E2F1 complex and consequently enhanced cell proliferation [21]. These observations demonstrated that SUMOylation can enhance pRb phosphorylation to promote cell cycle progression. As CDK4/6 have also been reported to initiate pRb phosphorylation at early G1 phase [67], CDK4/6 may also play a role in SUMOylation-mediated enhanced phosphorylation of pRb. On the other hand, SUMOylation of pRb is also capable of impeding cell cycle progression. For example, Kaposi’s sarcoma herpes virus latent protein LANA2 interacted with pRb via its LXCXE sequence motif, and hindered pRb SUMOylation to bypass cell cycle G1 arrest [68]. In addition, SUMO-2/3 modified pRb in HEK293 cells stably expressing SUMO-2/3, and the SUMOylation of pRb resulted in cell cycle arrest [69]. It is notable that SUMOylation of pRb by distinct SUMOs generates different effects in cell cycle regulation. The specific signaling pathways involved in recruiting specific SUMOs to modify pRb to exercise different functions in diverse contexts remain to be investigated.

Thus far, not many E3 ligases have been documented to be engaged in pRb SUMOylation. Only one study reported that, in an in vitro SUMOylation assay, pRb was SUMOylated specifically with SUMO-2/3 by the SUMO E3 ligase K-bZIP, which is encoded by the Kaposi’s sarcoma-associated herpesvirus (KSHV) [70]. Nonetheless, it remains unknown which site on pRb was SUMOylated by K-bZIP, as well as the in vivo functional outcomes of this SUMOylation.

### (B) DeSUMOylation of pRb

SUMOylation of pRb can be reversed via SENPs by removing SUMOs. In mouse embryonic fibroblasts, the SUMO protease SENP1 associated with and deSUMOylated pRb via removing SUMO-1 [71]. The pRb deSUMOylation decreased its proteasomal turnover and consequently repressed E2F1 activity, leading to blockage of S phase entry and reduced cell proliferation [71]. This finding confirms that SUMOylation can serve as a molecular modulator governing pRb stability and cell cycle regulation. Notably, markedly decreased SUMOylation of pRb and increased expression of SENP1 were observed in keratinocytes from lesions of vitiligo in comparison with normal keratinocytes, suggesting deSUMOylation of Rb in keratinocytes might play a role in the pathogenesis of vitiligo [72].

### (A) Phosphorylation of pRb

Protein phosphorylation is catalyzed by protein kinases to add phosphate groups from ATP or other nucleoside phosphates to amino acids of protein substrates [73]. In eukaryotic cells, phosphorylation at serine, threonine and tyrosine residues of the protein substrates by protein kinases accounted for around 84%, 15%, and < 1% respectively of the total protein phosphorylation events studied by phosphoproteomics [74]. This modification may occur on a single site or on multiple sites of the same substrate protein molecule [75]. In human, more than 500 protein kinases have been identified to regulate protein phosphorylation and most of them are serine/threonine kinases [76]. These protein kinases contain several conserved motifs in protein structures including an ATP binding domain, activation loop and catalytic domain [77]. Protein phosphorylation mediated by these kinases acts as a molecular switch to regulate a plethora of protein functions, involving protein turnover, enzymatic activity, protein–protein interaction, protein conformation alteration and localization, which in turn affect numerous cellular functions such as cell growth and differentiation [78]. Protein phosphorylation is reversible by removing the phosphate groups from the protein substrates by the phosphatase (PP) enzymes [79]. Phosphatase 1 (PP1) and phosphatase 2A (PP2A) are the two major phosphatases, and both of them account for more than 90% of protein phosphatase activity in eukaryotes [80]. Protein kinases and phosphatases work in dynamic balance to regulate protein functions.

Phosphorylation of pRb at multiple sites induces site-specific and diverse global conformational changes to modulate pRb functional outputs via affecting its interaction with E2F and LXCXE motif containing proteins (Table 3 and Fig. 1C). For example, phosphorylation at T821/T826 by CDK2-cyclin A or CDK4-cyclin D1 impeded pRb interaction with LXCXE motif containing viral oncoproteins by promoting an intramolecular interaction between pRbC and the pocket B domain [10, 81, 82]. Additionally, pRb phosphorylation at both S608/S612 and T356/T373 was necessary for inhibiting the interaction between the E2F^TD^ and the pRb pocket domain, where S608/S612 phosphorylation promoted an intramolecular association between the flexible pocket linker and the pocket domain while T373 phosphorylation led to an intramolecular interaction between pRbN and the pocket domain [83, 84]. Strikingly, S788/S795 and S807/S811 phosphorylation of pRb disrupted the interaction between the pRbC^N^ and the E2F^MB^-DP^MB^ heterodimer, and also the interaction of pRb pocket domain and E2F^TD^ [10, 85]. These dual effects might be explained by an intramolecular association between the pRbC and the pocket domain, even though S807/S811 phosphorylation did not contribute to inhibition of pRb and E2F^TD^ interaction [85]. In contrast, S807/S811 sites on pRb might serve as priming sites to promote an intermolecular association to facilitate further phosphorylation events [86].

**Table 3:** Summary of phosphorylation events in pRb with reported functional outcomes

Functional outcomes	Phosphorylated sites	Kinases	References
Impede pRb interaction with LXCXE motif containing proteins	T821/T826	CDK2 and CDK4	[10, 81, 82]
Hinder pRb–E2F^TD^ binding and/or pRb–E2F^MB^–DP^MB^ binding	S608/S612 and T356/T373	Unknown	[83, 84]
	S788/S795 and S807/S811		[10, 85]
Promote G0 exit and G1 entry	S807/S811	p38γ MAPK	[88]
		CDK3	[87]
Abrogate pRb growth suppression activity and promote cell cycle progression	S780	CDK4	[94]
	S795	CDK4	[93]
	S567	CDK2	[95]
	S807/S811	CDK5	[96]
	S780, S795 and S807/S811	CDK4	[97]
	S608	Raf-1	[98, 99]
	S807/S811, S780 and T821/T826	p38γ MAPK	[88]
	S780, S807/S811 and T821	UL97	[100]
Delay S phase progression	S612	BGLF4 kinase	[101]
Transit G2/M phase or exit cell cycle	S804	AMPK	[102]
Increase E2F transcriptional activities and trigger cell death	S795	CDK4/6	[103, 104]
	S780, S795 and S807/S811	CDK5	[105]
	S780	p38 MAPK	[106]
	S567	p38 MAPK	[107]
	T821	SAPK/JNK	[108]
Promote cell survival	S249/T252	p38α MAPK	[109]
	Y805	Abl	[110]
	S612	Chk1/2	[111]
	S807	Unknown	[177]
Hinder pRb and HDAC5 interaction	S249/T252 and T821	CDK4/6 and CDK2	[112]
Promote cancer immunity	S249/T252	CDK4/6	[113]
Enhance chromatin decondensation	S838/T841	p38 MAPK	[114]
Promote pRb–E2F1 binding to negatively modulate endoreduplication and avoid polyploidy formation	S780	Aurora B kinase	[115]
Suppress tumourigenesis	T821	CDK4/6	[176]

Site-specific phosphorylation on pRb plays crucial roles in cell cycle regulation (Table 3). During the G0/G1 transition of the cell cycle, cyclin C-CDK3 phosphorylated pRb at S807/S811 to promote efficient G0 exit and subsequent G1 phase entry [87]. Additionally, p38γ MAPK also phosphorylated pRb at S807/S811 to regulate entry into the cell cycle [88]. In early G1 phase, cyclin D-CDK4/6 hypophosphorylated pRb to activate pRb, which allowed for tightly governing the length of G1 phase, continued binding with E2F and repression of its activity [89–91], while cyclin E-CDK2 hyperphosphorylated pRb in late G1 phase to dissociate pRb with E2F and enhance E2F transcriptional activities [89]. During G1/S phase transition, cyclin D-CDK4/6 docked at the C-terminal helix of pRb and mediated site-specific phosphorylation of pRb to facilitate dissociation of pRb from chromatin and activation of E2F1 transcriptional activities [92]. For example, cyclin D1-CDK4 phosphorylated pRb at S780 or S795 to dissociate E2F1 from pRb and abrogate pRb growth suppression activity [93, 94], while cyclin E-CDK2 phosphorylated pRb at S567 within the pocket domain, resulting in disrupted interaction of pocket domain A and B and consequent E2F dissociation [95]. Interestingly, pRb was also targeted by CDK5 for phosphorylation at S807/S811, leading to expression of the E2F-responsive genes and enhanced cell cycle progression [96]. Moreover, cyclin Y-CDK4 phosphorylated pRb at S780, S795 and S807/S811, leading to pRb inactivation, enhancement of E2F transcriptional activities and G1/S phase transition [97]. In addition to CDK-mediated pRb phosphorylation during G1/S phase transition, other kinases engaged have also been observed. For example, Raf-1 kinase phosphorylated pRb at S608 and inactivated it to promote E2F1 transcriptional activities and cell cycle progression [98, 99]. p38γ MAPK sensitized pRb phosphorylation and inactivation by CDKs at several known CDK target residues (S807/S811, S780 and T821/T826) to promote cell cycle progression [88]. Human cytomegalovirus (HCMV) UL97 protein with kinase activities phosphorylated pRb at S780, S807/S811 and T821 and thus inactivated pRb, contributing to cell cycle progression [100]. Intriguingly, pRb phosphorylation at S612 by BGLF4 kinase disturbed DNA synthesis and delayed S phase progression [101]. 5ʹ-AMP-activated protein kinase (AMPK), independent of CDK4/6, directly phosphorylated pRb at S804 (S811 in human) to transit G2/M phase or exit cell cycle [102]. Taken together, these studies indicate that functions of pRb in cell cycle are tightly regulated via site-specific phosphorylation by distinct kinases.

Phosphorylation of pRb regulates its pro-apoptosis or pro-survival functions in a cell cycle-independent fashion. On one hand, site-specific pRb phosphorylation sequesters pRb from E2F, increases E2F transcriptional activities and triggers cell death. For example, CDK4/6-mediated phosphorylation of pRb at S795 enhanced E2F/DP transcriptional activities, leading to neuron apoptosis in the context of DNA damage or beta-amyloid accumulation [103, 104]. Under pathological conditions, CDK5 with enhanced activity cooperated with its co-activator p35 directly promoted pRb phosphorylation at S780, S795 and S807/S811, and increased E2F transcriptional activities to trigger apoptosis of neurons [105]. In response to Fas stimulation, Gadd45b promoted p38 MAPK-mediated phosphorylation of pRb at S780, leading to cell apoptosis [106]. Under genotoxic stress, p38 MAPK directly phosphorylated pRb at S567 independent of CDKs and triggered interaction between pRb and the human homolog of MDM 2, resulting in pRb degradation, dissociation of E2F1 and eventually cellular apoptosis [107]. SAPK/JNK was activated upon γ-irradiation (IR) and phosphorylated pRb at T821, which resulted in IR-induced apoptosis [108]. On the other hand, pRb phosphorylation also promotes cell survival independent of cell cycle under stress conditions. For example, p38α MAPK phosphorylated pRb at S249 and T252 to enhance pRb binding affinity toward E2F1 and retard expression of E2F-responsive genes, which led to S-phase entry delay and an increase in cell survival under stress conditions [109]. Abl kinase specifically phosphorylated pRb at Y805 to partially reduce apoptosis induced by excess level of pRb expression in Abl-dependent tumour cells [110]. Checkpoint kinases 1/2 (Chk1/2) phosphorylated pRb at S612 and enhanced pRb–E2F1 complex formation, which promoted cell survival and the anti-apoptotic activity of pRb in response to DNA damages [111]. Collectively, these studies demonstrate that the negative or positive apoptosis functions of pRb are modulated by distinct kinases via site-specific phosphorylation in a context-dependent manner.

Aside from involvement in regulation cell cycle and apoptosis, pRb is also engaged in several less known processes, including regulation of transcription and cancer immunity, chromatin decondensation and cell mitosis. Phosphorylation on pRb S249/T252 by CDK4/6 and T821 by CDK2 hindered pRb and HDAC5 interaction, which attenuated transcriptional repression activities of pRb [112]. Notably, S249/T252 phosphorylation by CDK4/6 enhanced pRb interaction with nuclear factor κB (NF-κB) protein p65, which regulated expression of a subset of NF-κB target genes and suppressed programmed death ligand-1 (PD-L1) expression to promote cancer immunity [113]. p38 MAPK phosphorylated pRb at S838/T841 upon mimetic T-cell receptor activation, which interfered with the interaction of pRb and condensin II with chromatin and accelerated chromatin decondensation via release of pRb and condensin II from chromatin [114]. Aurora B kinase phosphorylated pRb at S780 in response to aberrant mitosis to promote pRb–E2F1 binding and hindered E2F1 promoter activation, thereby negatively modulating endoreduplication and avoiding polyploidy formation [115].

pRb is also phosphorylated by other kinases while the relevant functional outcomes have yet been determined. For example, pRb was phosphorylated by cell division cycle 2 (CDC2) kinase in mitotic cell extracts in vitro [116], whereas the phosphorylation sites and impacts on pRb functions particularly during mitosis warrant further investigation. Apoptosis signal-regulating kinase 1 (ASK1) was able to bind with pRb through its LXCXE motif and phosphorylated pRb efficiently in vitro with the phosphoacceptor residues and functional importance remaining unknown [117]. The effects of phosphorylation by these kinases on pRb functions, particularly in various contexts, require further studies.

### (B) Dephosphorylation of pRb

Two phosphatases, PP1 and PP2A, dephosphorylate pRb during cell cycle. PP1 dephosphorylated pRb by competing with cyclins-CDK for pRb binding through an overlapping docking site in the pRbC during mitotic exit and G1 entry [118–122]. Interestingly, PP1α, an isoform of PP1, dephosphorylated and activated pRb to delay G1-S transition, leading to cell growth arrest [123]. PP1 and PP2A also dephosphorylate pRb in response to distinct cellular stresses. For example, in Ras-induced cellular senescence, PP1α associated with NORE1A and further interacted with pRb, leading to pRb dephosphorylation and arrest of cell cycle progression [124]. PP1-mediated dephosphorylation of pRb was hindered by PP1 nuclear targeting subunit (PNUTS) through blocking their binding sites on PP1 [125]. PNUTS depletion promoted PP1-mediated dephosphorylation of pRb, leading to dissociation of E2F1 from pRb and apoptosis [126–128]. In diploid S phase cells with DNA damages, PP2A dephosphorylated pRb and relocated hypophosphorylated pRb to selected DNA replication control sites to contain the abnormal post-damage re-replicative activity [129]. Notably, prolyl isomerase Pin1 could inhibit PP2A activities to enhance hyperphosphorylation of pRb to impede S phase checkpoint upon S-phase DNA damages [130]. Additionally, PP2A dephosphorylated pRb at T356, T821 and T826, giving rise to S-phase DNA synthesis reduction under oxidative stress [131]. Dephosphorylation of pRb at T821 was found necessary for apoptosis induction [132], and T821 on pRb is a target of PP2A but not PP1 [122, 131], hence future studies should confirm if PP2A plays a role in dephosphorylation of pRb at T821 to mediate apoptosis.

### (A) Acetylation of pRb

Acetylation can be catalyzed by enzymes or non-enzyme reactions (reactive acetyl derivatives) through transferring an acetyl group to the ε-amino groups of lysine residues on protein substrates [133]. The enzymatic acetylation is conducted by lysine acetyltransferases (KATs), while non-enzymatic acetylation requires reactive acetyl derivatives including acetyl-CoA, acetylphosphate and acetyladenylate [134–136]. KATs are classified into three major families: the p300/CBP family, the MYST family and the Gcn5-related *N*-acetyltransferase (GNAT) family [137]. A variety of proteins can be modified by KATs, including histones, transcription factors and nuclear import factors, to regulate numerous diverse cellular events involving DNA recognition, protein stability and protein–protein interaction [138]. Acetylation is reversible and the acetyl groups can be removed from the substrate molecules by lysine deacetylases (KDACs) [139]. Two families of KDACs have been identified and can be further divided into four classes: the NAD^+^-dependent sirtuin family (class III) [140] and the zinc-dependent Rpd3/Hda1 family (class I, II and IV) [141]. Deacetylation of proteins has been shown to play essential roles in biological processes including DNA repair, cell fate determination and metabolic regulation [142].

Acetylation of pRb modulates its phosphorylation, protein–protein interaction and control of gene transcription. Initially pRb was found to be acetylated at K873/K874 by p300/CBP protein with the histone acetyltransferase (HAT) activity during cell cycle progression and cell differentiation [22]. The K873/K874 acetylated pRb impeded its phosphorylation by cyclin E-CDK2 but displayed a stronger binding affinity towards MDM2 than that of the non-acetylated pRb, whereas the acetylation of pRb exerted no impact on the association of its pocket domain with E2F1 [22]. As K873/K874 on pRb are parts of the CDK-docking sites [143], it is necessary to explore how K873/K874 acetylation obstructs pRb phosphorylation by cyclin E-CDK2. While in response to etoposide-mediated DNA damage, pRb was also acetylated on K873/K874 and the DNA damage-induced pRb acetylation hindered the association of E2F1 and pRbC [144], which is distinct from the observation that pRb acetylation at K873/K874 did not influence E2F1–pRb pocket domain interaction [22], suggesting the context-dependent acetylation has distinct effects on pRb–E2F1 binding. Additionally, upon adenovirus infection, E1A sequestered pRb from E2F transcription factor and enhanced p300-mediated K873/K874 acetylation of pRb, acetylated form of which formed a ternary repressing complex with p300 and E1A to condense chromatin, resulting in specific repression of host genes with high p300 association that interfered with efficient virus infection, such as the TGFβ-, TNF-, and interleukin-signaling pathway components [145].

Acetylation of pRb affects its functions in regulating cell differentiation. During cell differentiation, pRb was acetylated at K873/K874 by p300-Associated Factor (P/CAF) and P/CAF could cooperate with p300 to enhance the pRb acetylation, which exerted no impact on pRb-mediated acute cell cycle arrest or repressive function of pRb on E2F transcriptional activities [146]. Nonetheless, the acetylated pRb collaborated with MyoD transcription factor for permanent cell cycle exit and induction of late differentiation gene expression, where acetylated pRb increasingly associated with MDM2 to promote degradation of the MyoD repressor E1A-like inhibitor of differentiation (EID-1) [146]. As acetylated pRb showed stronger binding affinity toward MDM2 during cell differentiation [22, 146], it would be interesting to identify its other potential binding partners and the relevant biological effects. Notably, in differentiating human keratinocytes, pRb acetylation at K873/K874 was mediated by nuclear P/CAF but not p300 without affecting either the repression activity of pRb on E2F1 or pRb stability [147]. Instead, the acetylated pRb translocated into the nucleus to maintain the differentiated state of keratinocytes [147]. Although K873/K874 acetylation of pRb has been shown to be mediated by both p300 and P/CAF [22, 146], the major mediator of the pRb acetylation in cell differentiation appears to be P/CAF. Further investigations are warranted to confirm the principal role of P/CAF in pRb acetylation in cell differentiation.

Acetylation of pRb modulates its protein stability in a context-dependent manner. For example, HPV16 E7 oncoprotein engaged in p300/CBP-mediated pRb acetylation to destabilize pRb, where the full-length E7 dimer formed a ternary complex with pRb and the TAZ2 domain of p300/CBP to promote pRb acetylation and its subsequent degradation [148]. Besides, upon genotoxic damages induced by melphalan, tumour suppressor p14^ARF^ blocked HAT Tip60-mediated acetylation of pRb on its C-terminus and promoted accumulation of hypoacetylated pRb, preventing pRb from subsequent proteasomal proteolysis and therefore stabilizing pRb [149], which indicated destabilization of pRb due to acetylation. However, the exact lysine residues on pRb acetylated by Tip60 have not been identified while they should not be K873/K874 as acetylated by p300 and P/CAF. These studies suggest acetylation promotes pRb protein turnover in a context dependent manner.

### (B) Deacetylation of pRb

Deacetylation of pRb promotes its phosphorylation to modulate its functions in cell cycle regulation. Deacetylation of pRb on lysine residues by deacetylase sirtuin1 (SIRT1) was first identified in an in vitro assay [150]. In cells arrested via contact inhibition or in cells with etoposide-induced DNA damages, pRb was increasingly acetylated, and the acetylation was in an inverse correlation with pRb phosphorylation and could be reversed by SIRT1 [150], suggesting acetylation might impede phosphorylation of pRb. Later, another study showed that enforced SIRT1 expression promoted pRb phosphorylation at S795 to suppress cellular senescence in human diploid fibroblasts [151]. Moreover, SIRT1-mediated deacetylation promoted pRb phosphorylation and E2F1-mediated S-phase entry in murine renal epithelial cells [152]. Also in haploinsufficiency-induced senescence (HIS) in BRCA1^mut/+^ human mammary epithelial cells, misregulation of SIRT1 increased acetylation of pRb and a decline in its phosphorylation, which resulted in pRb-dependent HIS premature senescence [153]. Taken together, these investigations demonstrate that acetylation hinders pRb phosphorylation to modulate its functions in cell cycle regulation.

### (A) Methylation of pRb

Methylation on the ε-amine of a lysine side chain of proteins was first identified in a bacterial flagellar protein and later in calf thymus histone proteins [154, 155]. This process is catalyzed by protein lysine (K) methyltransferases (PKMTs) to transfer a methyl group from the methyl donor, *S*-adenosylmethionine (SAM), to the terminal side-chain ε-amine of lysine residues of the protein substrate [156]. The ε-amine of lysine residues can be modified with up to three methyl groups to generate three states of mono-, di- or tri-methylation with different functional outcomes depending on the recruitment of binding proteins [157]. Two groups of PKMTs have been identified. One is the SET-domain proteins which harbor a catalytic SET [SU(var)3–9, Enhancer of zeste and Trithorax] domain and mostly favor in targeting lysines in the flexible tails of histones. The other group is the seven-β-strand (7βS) family which can methylate a wide spectrum of substrates including proteins, lipids and nucleic acids [158, 159]. PKMTs are characterized to “write” while lysine demethylases (KDMs) and effector proteins to “erase” and “read” lysine methylation, respectively [160]. Functionally, lysine methylation plays substantial roles in protein–protein interaction [161], protein stability [162], protein subcellular localization [163] and protein–DNA interaction [164]. Similar to most of the PTMs, lysine methylation is reversible and the methyl groups on lysine residues can be removed by KDMs to reach a dynamic balance [165].

pRb is methylated by several methyltransferases to modulate its functions in cell cycle progression regulation, differentiation and DNA damage response (Table 4). Under etoposide induced DNA damage, pRb was highly methylated at K810 by Set7/9 methyltransferase, which antagonized phosphorylation at sites throughout the pRb protein to maintain pRb in a hypophosphorylated active state, suppressing E2F1 transcriptional activities and consequently arresting cell cycle progression [166]. Interestingly, Set7/9 also methylated pRb at K873 both in vitro and in vivo without DNA damage, which facilitated repression of E2F1-responsive gene expression and consequently led to cell cycle arrest [167]. Besides, the K873-methylated pRb interacted with heterochromatin protein 1 (HP1) in a Set7/9 dependent fashion to enhance the suppressive effects of pRb on E2F1 transcription activities and promote cell differentiation [167]. It is unclear if K873 methylation by Set7/9 obstructs pRb phosphorylation for suppressive activity on E2F1 transcription activities. Methylation of pRb at K810 was also mediated by SMYD2 in unperturbed cells to promote pRb phosphorylation at S807/S811, which enhanced E2F1 transcriptional activities and further accelerated cell cycle progression [168]. Nonetheless, the effect of K810 methylation on pRb by SMYD2 differs from that mediated by methyltransferase Set7/9 under etoposide-mediated DNA damage, suggesting that under different conditions, the K810 residue on pRb can be methylated via various methyltransferases to affect its phosphorylation and lead to different functional outcomes. Furthermore, SMYD2 also mono-methylated pRb at K860 during cell cycle re-entry, in cell differentiation and DNA-damage response, and mono-methylation of pRb at K860 facilitated its direct interaction with the methyl-binding domain of the transcriptional repressor L3MBTL1 [169]. As L3MBTL1 is able to associate with and inhibit the transcription of some E2F1 target genes [170], future studies are warranted to test the possibility that SMYD2-mediated pRb mono-methylation might recruit it to the promoters of specific pRb/E2F target genes to repress their transcription.

**Table 4:** pRb methylation and relevant functional outcomes

Site	Methyltransferases/effector proteins	Functional outcomes	References
K810	Set7/9	Suppress E2F1 transcriptional activity and arrest cell cycle progression	[166]
	SMYD2	Enhance E2F1 transcriptional activity and accelerate cell cycle progression	[23]
	53BP1	Facilitate pRb to integrate cell cycle control and DNA damage response	[171]
	PHF20L1	Negatively regulate E2F-responsive genes for pRb-mediated G1/S checkpoint control	[172]
K873	Set7/9	Repress E2F1-responsive gene expression, arrest cell cycle progression and promote cell differentiation	[167]
K860	SMYD2	Facilitate pRb binding with L3MBTL1	[169]
R787	PRMT4	Disrupt pRb binding with E2F1, enhance E2F1 transcriptional activation and promote cell cycle progression	[173]

Some effector proteins have recently been identified to read lysine methylation on pRb. For example, in response to DNA damage, p53 binding protein 1 (53BP1) was recruited and specifically read K810 di-methylated pRb via its tandem tudor domain to act on the promoters of E2F-target genes, facilitating pRb to integrate cell cycle control and DNA damage response [171]. In contrast, in unperturbed cells, tudor-domain protein PHD-finger protein 20-like 1 (PHF20L1) selectively interacted with pRb and read pRb K810 mono-methylation by recruiting the MOF acetyltransferase complex [172]. The PHF20L1-MOF complex along with K810 mono-methylated pRb acted on E2F-responsive promoters, where MOF acetyltransferase complex might negatively regulate E2F-responsive genes for pRb-mediated G1/S checkpoint control [172].

Apart from lysine methylation, arginine residues on pRb have also been shown to be methylated. For example, protein arginine methyltransferase 4 (PRMT4) interacted with pRb and methylated several arginine residues on pRbC, among which some arginine residues were found methylated in vitro, such as R775, R787 and R798, while only R787 was identified to be methylated in vivo [173]. The R787 arginine methylation of pRb promoted pRb phosphorylation at its C-terminal domain and disrupted its binding with E2F1, giving rise to E2F1 transcriptional activation and enhanced cell cycle progression [173]. Future works should further address if these arginine residues are methylated to modulate pRb functions under specific conditions.

### (B) Demethylation of pRb

Demethylation on pRb protein has also been reported. Jumonji domain containing 3 (JMJD3), a histone demethylase, interacted with pRb via its demethylase domain JmjC and demethylated pRb at K810, which hindered pRb interaction with CDK4 and decreased its phosphorylation at S807/S811 [174]. The resultant hypophosphorylated pRb repressed E2F target gene expression, contributing to cellular senescence and senescence-associated heterochromatin foci (SAHF) formation induced by activated oncogene H-Ras (H-RasV^12^) in human diploid fibroblasts WI38 [174]. This study provides further evidence that demethylation/methylation can crosstalk with phosphorylation of pRb protein at specific residues to modulate pRb functions.

## Conclusions and future perspectives

**Fig. 2:**
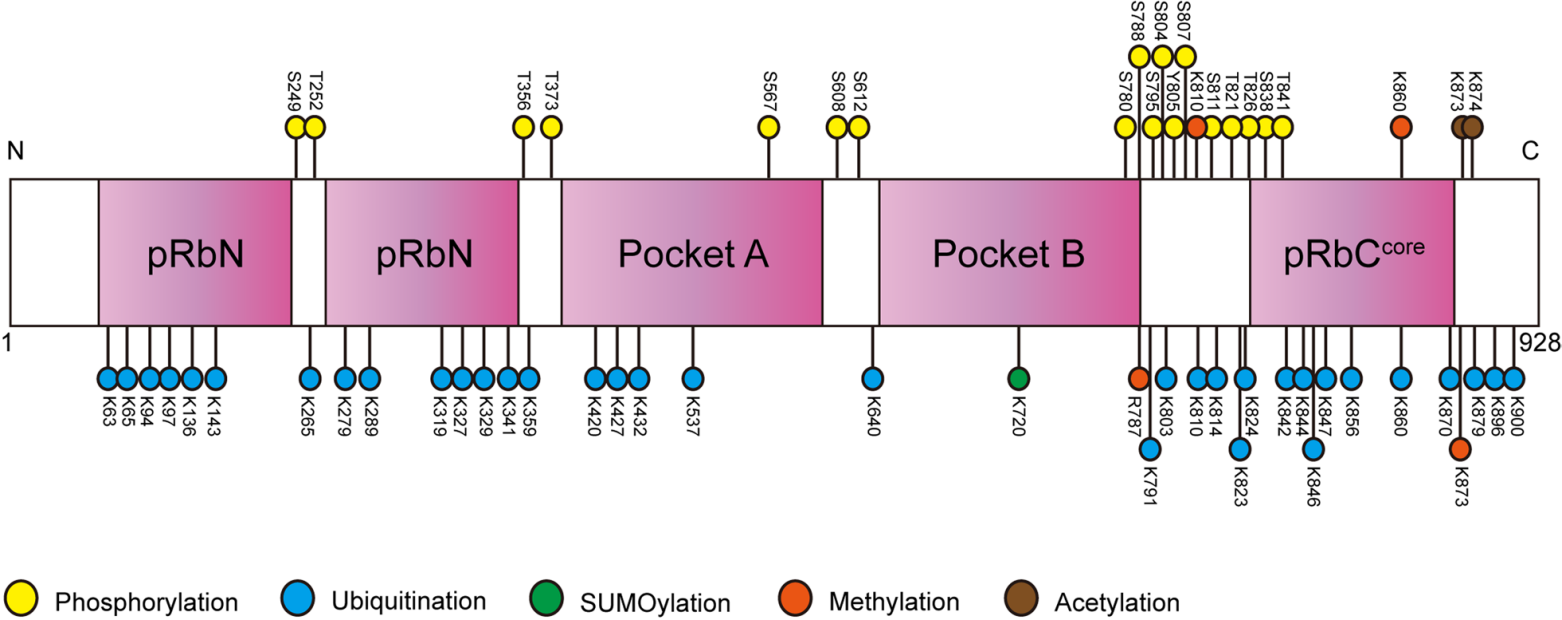
Summary of posttranslational modification sites on pRb. Sites of phosphorylation, SUMOylation, methylation and acetylation on pRb with reported functional outputs are shown, while sites of ubiquitination on pRb identified by mass spectrometry without documented functional outcomes are also shown. N and C indicate the N- and C-terminals of the protein

pRb is a multifunctional protein with impacts on cell proliferation, cell survival and differentiation. PTMs frequently modify pRb to regulate pRb functions through diverse mechanisms under different physiological and pathological settings (Fig. 2). Phosphorylation is the most extensively investigated form of PTMs modulating pRb functions in cell cycle regulation, whereas there are still many intriguing effects of pRb phosphorylation to be verified. For example, proteomics analyses revealed that pRb functions and its interactions with specific sets of proteins in G1-phase cells are finely modulated through mono-phosphorylation on any one of the 14 sites, indicating distinct transcriptional outputs beyond the E2F regulation [175]. Under different cellular contexts, these phosphorylated forms of pRb might play important roles in regulating various cellular processes. Furthermore, hyperphosphorylated pRb can also have inhibitory effects on tumourigenesis. T821 hyperphosphorylation of pRb unexpectedly suppress tumourigenesis via the inhibition of mTORC2-mediated activation of Akt partly through pRb binding with Sin1 [176]. This finding provides new information that pRb hyperphosphorylation can not only lead to uncontrolled cell proliferation and carcinogenesis but also might give rise to tumour suppression. Additionally, although pRb is widely accepted as a nuclear protein, its presence in mitochondria has also been reported and S807 phosphorylation of pRb in mitochondria protected C33A cells from apoptosis via association with Bax [177]. These intriguing new functions of pRb modulated by phosphorylation should be further validated and might lead to novel therapeutic targets for disease treatment in the future.

In comparison to phosphorylation, effects of other PTMs, including ubiquitination, SUMOylation, acetylation and methylation, on pRb functions are less understood. Many questions regarding the regulation of pRb functions by these PTMs remain to be addressed, particularly about the cellular contexts in which specific sites are modified and what complexes are formed or disrupted to execute relevant functions. In addition, it is unclear if there are any potential adaptor proteins that may bind to specific modification sites and engage in these modifications to influence pRb functions. Furthermore, competitions may exist between PTMs as some sites on pRb can be modified by different PTMs. K810 on pRb has been reported to be ubiquitinated in HEK293T cells despite the relevant functional outputs remain unclear [51]. This site can also be methylated by Set7/9 and SMYD2 as documented in other studies [23, 166]. Therefore, under specific contexts, one PTM might override another to play predominant roles in regulating pRb functions.

Studies on modulation of pRb functions by crosstalk of PTMs are also very limited thus far. PTMs may work together to synergistically regulate pRb functions in various cellular processes. SUMOylation enhanced pRb phosphorylation at early G1 phase to promote cell cycle progression [21]. Methylation also promoted pRb phosphorylation and therefore cell cycle progression [23]. On the other hand, PTMs may also antagonize one another under various cellular contexts. Methylation impeded pRb phosphorylation and thus cell cycle progression under DNA damaged conditions [166]. Different and diverse cellular contexts also account for the complicated crosstalk of PTMs to regulate pRb functions. Future studies should focus on proteomics and functional studies to explore the impacts of these PTMs and their crosstalk on pRb functions, which should provide opportunities for identifying novel targets for designing therapeutics to treat various human diseases with a higher specificity.

## Data Availability

Not applicable.
