# Correction: Post-translational modifications on the retinoblastoma protein

**DOI:** 10.1186/s12929-024-01079-6

**Published:** 2024-10-08

**Authors:** Linbin Zhou, Danny Siu‑Chun Ng, Jason C. Yam, Li Jia Chen, Clement C. Tham, Chi Pui Pang, Wai Kit Chu

**Affiliations:** 1 Department of Ophthalmology & Visual Sciences, The Chinese University of Hong Kong, Hong Kong, China; 2 Hong Kong Hub of Paediatric Excellence, The Chinese University of Hong Kong, Hong Kong, China; 3 Department of Ophthalmology & Visual Sciences, The Chinese University of Hong Kong, Hong Kong Eye Hospital, 147K Argyle Street, Kowloon, Hong Kong China

**Correction: Journal of Biomedical Science (2022) 29:33** 10.1186/s12929-022-00818-x

After publication of this article [1], it was brought to our attention that funding section need to be adjusted to:

Funding

This work was supported by the Health and Medical Research Fund, The Food and Health Bureau, The Government of the Hong Kong Special Administrative Region (07180306 and PR-HKCH-8 to J.C.Y).

The original publication has been updated.
